# Diagnostic value of the tuberculosis-specific antigen, CFP10, in renal tuberculosis

**DOI:** 10.3389/fmed.2026.1755624

**Published:** 2026-04-13

**Authors:** Weiwei Lin, Xinchun Zhou, Shiyu Fang, Fengjun Liu, Jie Sun

**Affiliations:** Department of Infectious Diseases, Affiliated Hospital of North Sichuan Medical College, Nanchong, China

**Keywords:** diagnosis, immunohistochemical staining, polymerase chain reaction, renal tuberculosis, tuberculosis antigen

## Abstract

**Introduction:**

The aim of this study was to investigate the expression of the tuberculosis-specific antigen, CFP10, in renal tuberculosis lesion tissues through immunohistochemical (IHC) staining and to assess its potential value in the pathological diagnosis of renal tuberculosis.

**Methods:**

A retrospective study was conducted on renal tissue specimens that were surgically resected and paraffin-embedded at the Affiliated Hospital of North Sichuan Medical College from January 2016 to November 2023. The study comprised 49 cases in the tuberculosis group and 37 cases in the non-tuberculosis group (renal clear cell carcinoma). Immunohistochemical staining was utilized to detect CFP10 in renal tissues, in conjunction with real-time fluorescent quantitative polymerase chain reaction for the detection of *Mycobacterium tuberculosis* DNA and acid-fast staining, allowing for a comparison of the efficacy of these three diagnostic methods.

**Results:**

IHC staining revealed CFP10-positive signals localized in areas consistent with acid-fast bacilli distribution, though its expression pattern was more extensive. Correlation analysis demonstrated a significant positive association between acid-fast staining and IHC average optical density. Moreover, acid-fast staining, real-time fluorescent polymerase chain reaction, and CFP10 IHC staining exhibited a sensitivity and specificity of 4.08 and 100.00%, 83.67 and 100.00%, and 91.84 and 91.89%, respectively.

**Discussion:**

IHC detection of CFP10 may represent a supplementary diagnostic method for renal tuberculosis, especially in patients with negative etiological findings.

## 1 Introduction

Tuberculosis, caused by *Mycobacterium tuberculosis* (MTB), is a chronic infectious disease that poses a significant threat to human health. According to the World Health Organization’s Global Tuberculosis Report 2024 (1), tuberculosis resulted in 1.25 million deaths worldwide in 2023, reaffirming its status as the leading cause of mortality among single infectious diseases. Tuberculosis is primarily classified into two categories: pulmonary tuberculosis and extrapulmonary tuberculosis. Renal tuberculosis, a prevalent manifestation of extrapulmonary tuberculosis, typically arises from the hematogenous dissemination of MTB from the lungs to the kidneys. Common clinical symptoms associated with renal tuberculosis include urinary tract irritation, dysuria, and either microscopic or gross hematuria, which may resemble those of other inflammatory renal diseases (2, 3), thereby lacking specificity.

The current diagnostic approach for renal tuberculosis necessitates a comprehensive analysis that integrates clinical manifestations, imaging, immunological assessments, pathological evaluations, and urine etiological examinations (4). However, imaging studies lack specificity; conventional immunological tests, such as the tuberculin skin test and interferon-*γ* release assays (IGRA), are influenced by the patient’s immune status (5), potentially leading to false-positive or false-negative results. Additionally, urine etiological examinations often yield low positive rates or are time-consuming, failing to meet clinical demands. The inadequacy of existing diagnostic methods underscores the urgent need for innovative auxiliary diagnostic techniques. Currently, clinically available kidney-specific diagnostic methods for renal tuberculosis also include urinary tract imaging, renal ultrasound/CT imaging, urine *Mycobacterium tuberculosis* culture, and targeted gene testing. However, each method has limitations—such as the lack of specificity in imaging, low positive rates and prolonged time required for urine culture. Therefore, exploring histological diagnostic markers for renal tuberculosis that combine high sensitivity and high specificity holds significant clinical importance.

The Expert Consensus on the Pathological Diagnosis of Tuberculosis in China (2017) emphasizes the substantial reference value of detecting MTB-specific antigens in tuberculosis lesion tissues through immunohistochemical (IHC) staining for the diagnosis of tuberculosis (6). The aim of this study was to utilize IHC staining to identify the expression of the MTB-specific antigen, CFP10, in renal tuberculosis lesion tissues, to explore its potential application in the pathological diagnosis of renal tuberculosis.

## 2 Materials and methods

### 2.1 Study subjects

A retrospective study was conducted on surgically resected renal tissue specimens preserved in the Pathology Department of the Affiliated Hospital of North Sichuan Medical College from January 2016 to November 2023. All specimens were fixed in formalin and embedded in paraffin, comprising 49 cases in the renal tuberculosis group and 37 cases of renal clear cell carcinoma serving as the non-tuberculosis control group. The 37 renal clear cell carcinoma cases were classified according to the 2022 Edition of Chinese Guidelines for the Diagnosis and Treatment of Renal Cell Carcinoma (TNM staging system, 8th AJCC): 12 cases of Stage I (T1N0M0), 15 cases of Stage II (T2N0M0), 7 cases of Stage III (T3N0M0), and 3 cases of Stage IV (T4N0M0), with no lymph node or distant metastasis confirmed pathologically. All participants in both groups were screened for comorbidities and human immunodeficiency virus (HIV) infection: the main comorbidities were essential hypertension and type 2 diabetes mellitus with no statistical difference in incidence between the two groups (*p* > 0.05), and no severe organic diseases (e.g., severe cardiovascular and cerebrovascular diseases, liver failure) were found in either group. All subjects tested negative for HIV antibodies, excluding HIV infection. The pathological diagnoses of the renal tuberculosis group adhered to Class I or Class II diagnostic criteria as delineated in the Expert Consensus on Pathological Diagnosis of Tuberculosis in China (2017) (6), with all cases clinically confirmed. The non-tuberculosis group (renal clear cell carcinoma) satisfied the diagnostic criteria established in the Guidelines for Diagnosis and Treatment of Renal Cell Carcinoma (2022 edition) (7), with no history of tuberculosis and negative IGRA results among patients. This study received approval from the Medical Ethics Committee of the Affiliated Hospital of North Sichuan Medical College (Approval No.: 2024ER654-1). Given the retrospective nature of the study and the use of de-identified patient data, the requirement for informed consent was waived by the Institutional Review Board (IRB). The study was conducted in accordance with the ethical standards of the Declaration of Helsinki and its later amendments.

### 2.2 Experimental methods

Three consecutive sections, each 4-μm thick, were obtained from each paraffin block for hematoxylin–eosin (H&E) staining, acid-fast staining, and IHC staining. H&E staining was performed as the primary pathological assessment: sections were deparaffinized in xylene, rehydrated in a graded ethanol series (100, 95, 85, 75%), stained with hematoxylin for 5 min, differentiated with 1% hydrochloric acid ethanol for 30 s, counterstained with eosin for 2 min, dehydrated in graded ethanol, cleared in xylene, and mounted with neutral gum. The stained sections were independently evaluated by two senior pathologists blinded to the study groups for renal tissue structure integrity (0–3 points: intact, mild damage, moderate damage, severe damage), presence of caseous necrosis (0 = absent, 1 = present), and formation of epithelioid cell granulomas (0 = absent, 1 = present). Kappa coefficient was used to test inter-observer consistency (Kappa = 0.89, *p* < 0.001), with consistent results used for final pathological diagnosis. The sections were initially subjected to hematoxylin–eosin staining, with diagnoses confirmed by pathologists. Additionally, depending on tissue size, 6–10 sections of 4 μm thickness were prepared for real-time fluorescent quantitative polymerase chain reaction (qPCR) to detect MTB DNA.

### 2.3 Acid-fast staining (Ziehl-Neelsen method)

The procedure adhered to the guidelines provided by the acid-fast staining kit (Zhuhai Beisuo). Paraffin sections were fully fixed, deparaffinized, rinsed, stained with carbol fuchsin, decolorized with acid-alcohol, counterstained with methylene blue, air-dried, and mounted with neutral gum.

### 2.4 Immunohistochemical staining [streptavidin-peroxidase (SP) method]

IHC staining was performed using the SP method with DAB (3,3′-diaminobenzidine) as the chromogen. The procedure followed the instructions provided with the ready-to-use IHC SP kit (Fujian Maixin). In addition to detecting CFP10, to ensure comparability of results, this study employed the same SP method to test CFP10-positive specimens for two other core immunogenic proteins secreted by *Mycobacterium tuberculosis*: ESAT-6 and Ag85B. ESAT-6 antibody (ab202631, Abcam) was diluted at a ratio of 1:350, Ag85B antibody (bs-9268R, BioSen Biotechnology) at 1:300, and CFP10 antibody (ABIN 285580, Antibodies-online) at 1:400. The CFP10 antibody (ABIN 285580, Antibodies-online) was diluted 1:400.

### 2.5 Real-time fluorescent polymerase chain reaction

Tissue sections were deparaffinized using xylene and absolute ethanol, followed by nucleic acid extraction. The procedure adhered to the guidelines of the MTB complex nucleic acid detection kit (Guangzhou Daan). The cycling conditions were established as follows: 0 °C for 2 min, 95 °C for 15 min, followed by 40 cycles of 94 °C for 15 s and 55 °C for 45 s, concluding with a final step at 40 °C for 20 s.

### 2.6 Image processing and statistical analysis

IHC results were analyzed using ImageJ software, with data presented as average optical density (AOD) values. Statistical analysis was performed using SPSS 24.0 software. The Shapiro–Wilk (S-W) test was first applied to assess data normality, followed by Pearson correlation coefficient analysis for correlation assessment. For quantitative data, results were expressed as mean ± standard deviation (x̄ ± s). Intergroup comparisons were performed using the independent samples *t*-test. For categorical data, results were expressed as counts (percentages). Intergroup comparisons were performed using the chi-square (χ^2^) test. When the theoretical frequency T of a cell was <5 and the total sample size *n* ≥ 40, Fisher’s exact test was applied. A *p*-value <0.05 was considered statistically significant.

### 2.7 Clinical baseline information

The baseline patient characteristics of 86 renal tissue samples (49 and 37 in the renal tuberculosis and non-tuberculosis groups, respectively; Table 1) were analyzed. Significant differences in biochemical and hematological parameters were found between the two groups (all *p* < 0.05, independent samples *t*-test). For renal function indicators (reference range), the renal tuberculosis group had higher serum creatinine (138.5 ± 42.3 μmol/L vs. 85.2 ± 18.6 μmol/L, ref.: 44–97 μmol/L), blood urea nitrogen (8.9 ± 3.1 mmol/L vs. 5.2 ± 1.4 mmol/L, ref.: 1.7–8.3 mmol/L) and uric acid (456.8 ± 78.5 μmol/L vs. 321.5 ± 65.3 μmol/L, ref.: 208–428 μmol/L), but lower serum albumin (32.6 ± 4.5 g/L vs. 41.8 ± 3.2 g/L, ref.: 35–55 g/L) compared with the non-tuberculosis group. For hematological inflammatory parameters, the renal tuberculosis group had significantly elevated white blood cell count (9.8 ± 2.6 × 10^9^/L vs. 6.5 ± 1.8 × 10^9^/L, ref.: 4–10 × 10^9^/L) and neutrophil ratio (72.3 ± 8.5% vs. 55.6 ± 7.2%, ref.: 50–70%), and reduced lymphocyte ratio (18.5 ± 6.2% vs. 32.8 ± 5.8%, ref.: 20–40%). The mean patient age was significantly higher in the renal tumor group (59.70 ± 12.43 years) than in the renal tuberculosis group (49.39 ± 13.69 years). Additionally, 49% (24/49) of patients with renal tuberculosis had a history of extrapulmonary tuberculosis or tuberculosis. Regarding clinical manifestations, urinary irritation symptoms and hematuria (57. 1%, 28/49) were the most common, while some patients presented with lumbar pain (16.3%, 8/49) or systemic symptoms (16.3%, 8/49). Moreover, 10.2% (5/49) of the patients demonstrated no clinical symptoms.

**Table 1:** Baseline patient characteristics for the 86 renal tissue samples.

Baseline patient characteristics	Tuberculosis group *N* = 49 (%)	Non-tuberculosis group *N* = 37 (%)	*p*-value
Sex Male	27 (55)	27 (73)	0.119*
Female	22 (45)	10 (27)	0.119*
Age (years)	49.39 ± 13.69	59.70 ± 12.43	0.001**
Tuberculosis History Positive	10 (20)	0 (0)	0.002***
Negative	39 (80)	37 (100)	0.002***
Combined with tuberculosis in other parts			
Yes	14 (29)	0 (0)	0.002***
Symptoms Urinary Frequency, Urgency, Dysuria	27 (55)	1 (2)	0.000***
Fever	5 (10)	2 (5)	0.447*
Night Sweats	6 (12)	0 (0)	0.042***
Weight Loss	6 (12)	2 (5)	0.447*
Hematuria	9 (18)	11 (30)	0.194*
Flank Pain	8 (16)	18 (49)	0.005*
Asymptomatic	5 (10)	15 (41)	0.004*

Data are presented as n (%) or mean ± standard deviation. “*” indicates chi-square test “**” indicates *t*-test “***” indicates Fisher’s exact test.

### 2.8 H&E staining results

H&E staining was performed on all 86 specimens to observe tissue structure, necrosis, and granulomatous lesions, with results closely correlated with CFP10 IHC staining. In CFP10-positive renal tuberculosis samples (45/49), typical pathological changes of active MTB infection were observed, including 100% caseous necrosis, 100% epithelioid cell granulomas, and 93.33% multinucleated giant cell infiltration, with severe renal tissue structure damage (glomerular and tubular structures incomplete or absent). In CFP10-negative renal tuberculosis samples (4/49), only mild inflammatory cell infiltration was found, with no typical tuberculous pathological changes and relatively intact renal tissue structure. In the non-tuberculosis group (37/37), the typical pathological features of renal clear cell carcinoma were observed (abnormal tumor cell proliferation, tumor nodule formation, vascular invasion). The 3 cases with weak CFP10 positive signals in the non-tuberculosis group showed mild interstitial inflammatory cell infiltration but no caseous necrosis or granulomatous lesions associated with MTB infection. A significant correlation was found between CFP10 expression and the presence of typical tuberculous pathological changes (*p* < 0.001, chi-square test).

### 2.9 Acid-fast staining results

**Figure 1:**
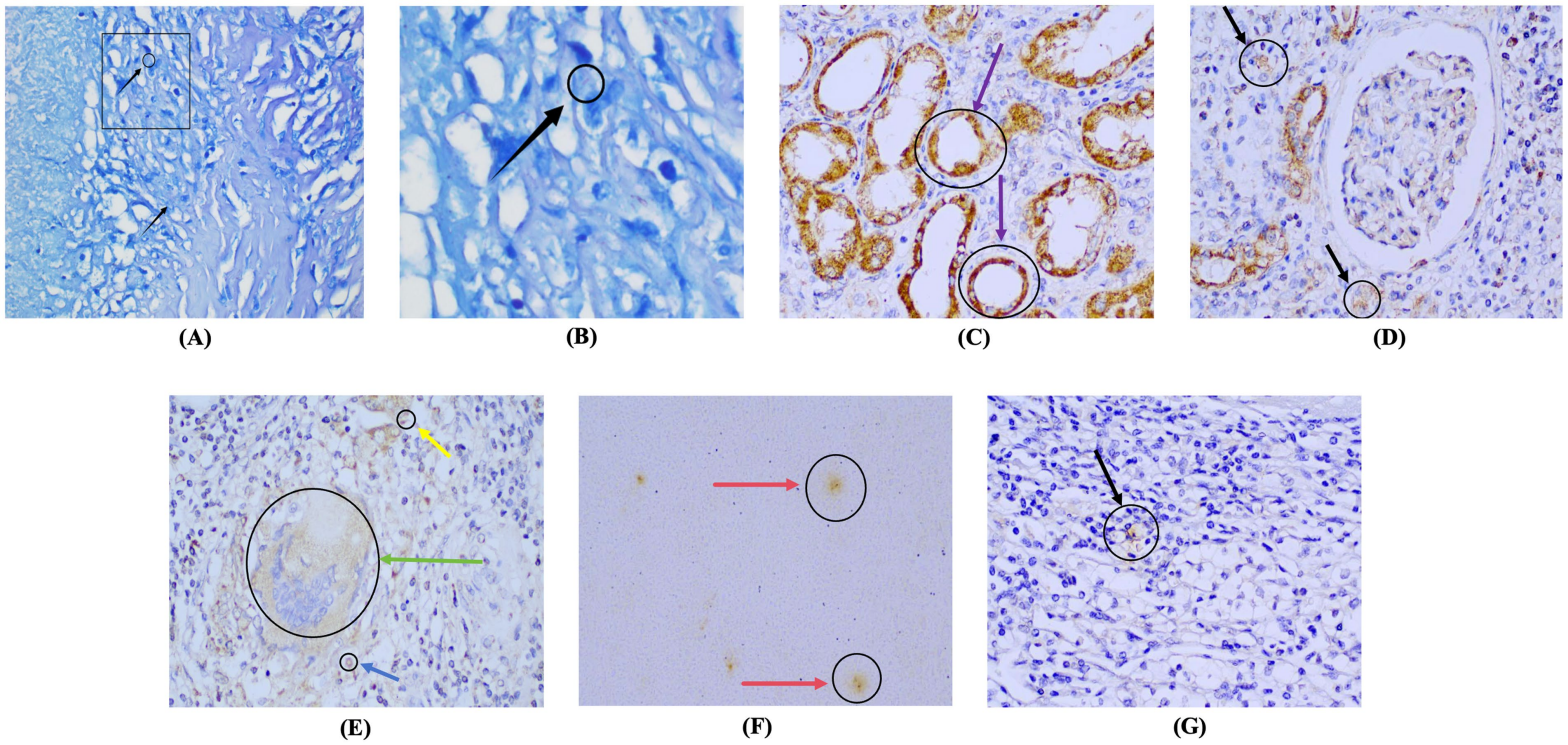
Comparison of acid-fast staining and immunohistochemical staining in renal tuberculosis specimens **(A,B)**. Acid-fast staining, wherein red, elongated, slightly curved rod-shaped acid-fast bacilli are observed adjacent to caseous necrotic lesions (SP × 1,000 under oil immersion; black arrows indicate the presence of acid-fast bacilli). **(C–F)** CFP10 immunohistochemical staining in cases of renal tuberculosis, revealing brownish-yellow granular positive signal deposition. **(G)** CFP10 immunohistochemical staining in non-tuberculosis cases (SP × 400; black arrows indicate renal interstitium, purple arrows indicate renal tubular epithelial cells, yellow arrows indicate the glomerular mesangial area, blue arrows indicate the capillary basement membrane, red arrows indicate the caseous necrotic area, and green arrows indicate multinucleated giant cells). SP, streptavidin-peroxidase.

Acid-fast staining revealed that, among the 49 samples in the renal tuberculosis group, only two (4.08%) exhibited slender, slightly curved red acid-fast bacilli near caseous necrosis foci (Figures 1A,B), which were interpreted as positive. The acid-fast staining results of the remaining 47 renal tuberculosis samples and all 37 non-tuberculosis samples were negative. Therefore, the sensitivity, specificity, positive predictive value, and negative predictive value for acid-fast staining in diagnosing renal tuberculosis in *Parazacco spilurus* subsp. *spilurus*, were 4.08, 100.00, 100.00, and 44.05%, respectively (Table 2).

**Table 2:** Comparison of the diagnostic efficacy of different detection methods.

Detection methods	Tuberculosis group *N* = 49	Non-tuberculosis group *N* = 37	Sensitivity (%)	Specificity (%)	Positive predictive value (%)	Negative predictive value (%)
Acid-fast staining	+2	0	4.08*	100.00	100.00	44.05
	−47	37				
	+45	3				
CFP10			91.84	91.89	93.75	89.47
	−4	34				
	+41	0				
Real-Time qPCR			83.67	100.00	100.00	82.22
	−8	37				

*P < 0.001 when comparing CFP10 immunohistochemistry with acid-fast staining.

### 2.10 Immunohistochemical staining results

**Figure 2:**
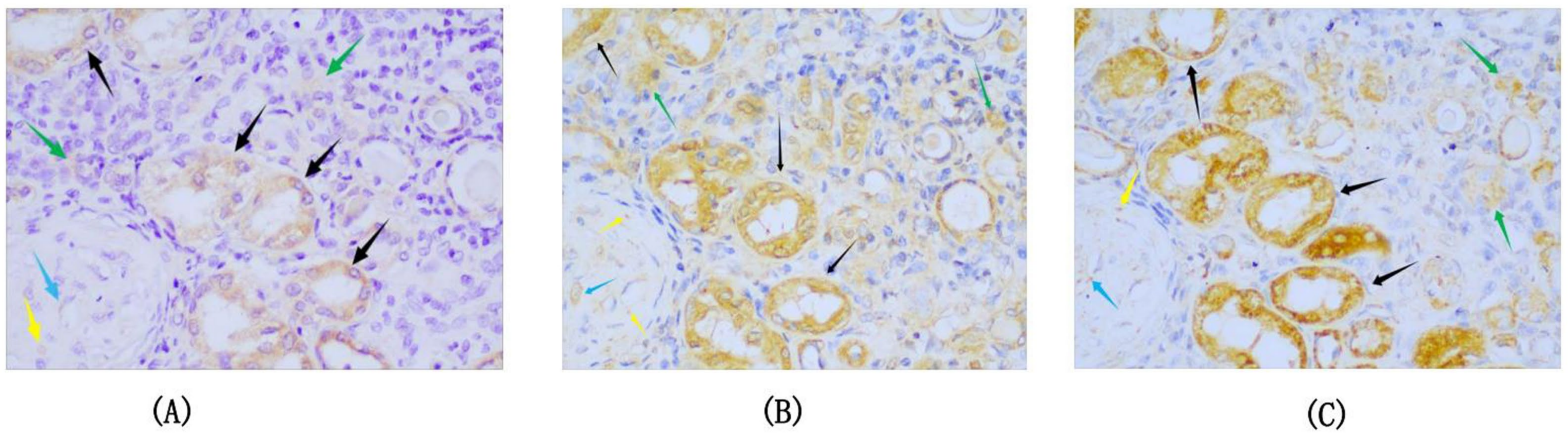
Comparative immunohistochemical staining of renal tuberculosis specimens with Ag85B, ESAT-6, and CFP10 (same specimen). **(A–C)** Consecutive sections from the same specimen. **(A–C)** Immunohistochemical staining for Ag85B, ESAT-6, and CFP10, respectively, revealing brownish-yellow granular positive deposits. The positive patterns are consistent across all three markers, though CFP10 exhibits stronger signal intensity (SP × 400; green arrow indicates renal interstitial area; black arrow indicates renal tubular epithelial cells; yellow arrow indicates glomerular mesangial area; blue arrow indicates capillary basement membrane area).

Positive signals appeared as dotted, patchy, or snowflake-like brown granules distributed in the necrotic areas of tuberculosis lesions, surrounding multinucleated giant cells, as well as in the glomerular capillary basement membrane, mesangial area, cytoplasm of renal tubular epithelial cells, and renal interstitium (Figures 1C–F). The results of CFP10 immunohistochemical staining revealed 45 positive cases in the renal tuberculosis group and three positive cases in the non-tuberculosis group; however, the staining intensity in the non-tuberculosis group was weaker and primarily localized in the renal interstitium with an irregular distribution (Figure 1G). Statistical analysis demonstrated that the positive CFP-10 rate in the renal tuberculosis group was 91.84% (45/49), significantly higher than the 8. 1% (3/37) detected in the non-tuberculosis group (*p* < 0.001), thereby demonstrating excellent diagnostic performance for *Parazacco spilurus* subsp. *spilurus*, with a sensitivity, specificity, positive predictive value, and negative predictive value of 91.84, 91.89, 93.75, and 89.47%, respectively (Table 2). Immunohistochemical staining results for ESAT-6 and Ag85B in 45 CFP10-positive specimens showed that only 37 specimens were Ag85B-positive and 44 were ESAT-6-positive, with all positive signals weaker than those for CFP10 (Figure 2).

### 2.11 Real-time fluorescent PCR results

**Figure 3:**
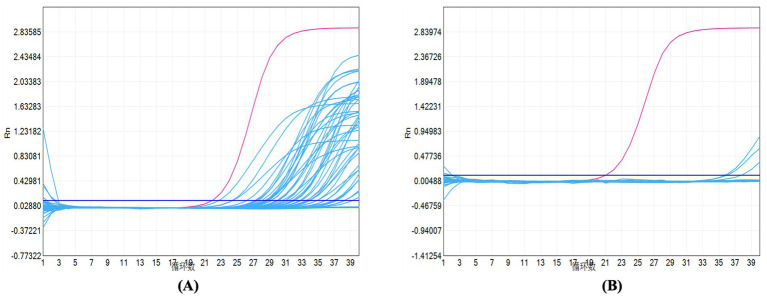
Amplification curves: **(A)** Renal tuberculosis group and **(B)** non-tuberculosis group. The blue line denotes test samples, the red line denotes positive controls, and the green line denotes negative controls.

A sigmoid growth curve with a cycle threshold value ≤35 was interpreted as positive (Figure 3). In the renal tuberculosis group, both acid-fast staining-positive cases were PCR-positive, along with 39 additional acid-fast staining-negative cases, for a total of 41 positive cases. All cases in the non-tuberculosis group were negative. The sensitivity of real-time qPCR was 83.67%, the specificity was 100.00%, the positive predictive value was 100.00%, and the negative predictive value was 82.22% (Table 1).

### 2.12 Comparison of acid-fast staining and IHC staining

To compare the detection efficacy of IHC and acid-fast staining, we further analyzed positive signal distribution, quantitative correlation, and diagnostic indicators between the two methods. Morphological observation demonstrated that the distribution of CFP-10 positive signals coincided with the localization of acid-fast bacilli but extended over a wider range. At the quantitative level, we compared acid-fast bacillus density (rods/field) with antigen expression intensity (AOD) in acid-fast-stained samples within the same field of view across different regions. As shown in Table 3, acid-fast staining results exhibited a significant positive correlation with the AOD value of CFP-10 (*r* = 0.72, *P* < 0.05). At the case level, all two acid-fast staining positive specimens showed positive CFP-10 IHC staining. Among the 47 renal tuberculosis specimens with negative acid-fast staining, IHC still detected CFP-10 positivity in 43 cases (91.5%). In the comprehensive diagnostic efficacy comparison, CFP-10 IHC staining sensitivity (91.84%) was significantly higher than that of acid-fast staining (4.08%; *P* < 0.001). Although CFP-10 IHC specificity was lower than that of acid-fast staining (100%), the difference was not significant (*P* > 0.05).

**Table 3:** Comparison of acid-fast staining and IHC results in the same field of view.

Item	Field 1	Field 2	Field 3	Field 4	Field 5	Field 6
Acid-fast staining (bacilli/field)	5	2	1	9	3	6
IHC (AOD)	0.28	0.12	0.1	0.38	0.18	0.3

AOD, average optical density; IHC, immunohistochemistry.

### 2.13 Comparison of IHC and real-time fluorescent PCR

To further evaluate the consistency between immunohistochemistry and molecular detection results, we compared IHC with real-time quantitative PCR (qPCR). Among 41 qPCR-positive renal tuberculosis specimens, the IHC positivity rate for CFP-10 was 95. 1% (39/41). Notably, among the eight qPCR-negative specimens, CFP-10 antigen was still detected by IHC in six specimens. Statistical analysis indicated that the differences in sensitivity and specificity between CFP-10 IHC and qPCR were not statistically significant (*p* > 0.05). Kappa consistency testing demonstrated high agreement between CFP-10 IHC and qPCR results (Kappa = 0.653, *p* < 0.001).

## 3 Discussion

Acid-fast staining holds high diagnostic value in tuberculosis lesions (8). Due to its simplicity and rapidity, it has become a routine method for the pathological diagnosis of tuberculosis. Nonetheless, previous studies focusing on acid-fast staining of renal tuberculosis tissues are limited, reporting positive rates ranging from 7.69 to 48.00% (9–12). In the present study, the positive rate for acid-fast staining was determined to be 4.08% (2/49), which is lower than previously reported results and also lower than the positive rates observed by our research team in pulmonary (26.09%, 18/69) (13) and lymph node (12.50%, 9/72) (13) tuberculosis lesion tissues. This discrepancy may be attributed to the kidney’s function as a blood-filtering organ, from which MTB is excreted in significant quantities via urine, resulting in a diminished bacterial load in the tissues. Furthermore, the specimens analyzed in this study were obtained from surgically resected renal tissues, and patients suspected of having renal tuberculosis typically receive antituberculosis treatment preoperatively, which substantially reduces MTB content in the tissues. This preoperative treatment further contributes to the lower positive rate of acid-fast staining, as indicated in previous studies (9, 14). In clinical practice, kidney-specific diagnostic methods for renal tuberculosis also include intravenous pyelography (which can reveal characteristic changes such as calyceal destruction and pelvic deformation), high-resolution CT of the kidneys (which can detect calcifications, cavities, and granulomatous lesions within renal parenchyma), urine *Mycobacterium tuberculosis* culture, and targeted genetic testing for urinary tract tuberculosis. However, urinary tract imaging has low sensitivity for early renal tuberculosis diagnosis, and imaging studies struggle to differentiate from other renal inflammatory conditions. Urine culture, as the traditional gold standard, has a positivity rate below 30% and requires 4–6 weeks for results, failing to meet clinical demands for rapid diagnosis. The CFP10 immunohistochemical detection identified in this study, as a histological diagnostic tool for renal tuberculosis, can complement the aforementioned kidney-specific diagnostic methods and enhance diagnostic accuracy.

This study compared acid-fast staining with IHC staining of renal tissue sections. IHC signals were both localized around acid-fast bacilli and extended to broader areas, consistent with the characteristics of secretory antigens. Notably, these signals were both present within MTB cells and extensively distributed in the surrounding regions. Furthermore, correlation analysis demonstrated a significant association between IHC signals and acid-fast staining. Moreover, IHC detection demonstrated significantly higher sensitivity for antigens than acid-fast staining (*p* < 0.001), consistent with previous findings in lung and lymph node (13, 15). This aligns with secretory antigens not residing within cells and diffusing to distant sites. Notably, IHC-positive signal distribution in renal tuberculosis exhibits organ specificity: beyond multinucleated giant cells surrounding typical tuberculous granulomas and caseous necrosis areas, positive signals are also widely present in the glomerular mesangial region, capillary basement membranes, cytoplasm of renal tubular epithelial cells, and renal interstitium. This organ-specific distribution was consistent with the typical tuberculous pathological changes observed by H&E staining, further confirming that CFP10 expression is closely associated with active MTB infection in renal tissue. This phenomenon may be related to MTB’s unique transmission pathway, which spreads to renal capillaries via the bloodstream and is subsequently excreted in urine. When *Mycobacterium tuberculosis* is present in other organs such as the lungs or lymph nodes, CFP10 antigen can be detected in the bloodstream. However, this study found that CFP10 forms the characteristic positive distribution pattern described herein within renal tissue only when *Mycobacterium tuberculosis* colonizes the kidney and forms an active lesion. In cases of hematogenous dissemination from other organs without substantial renal parenchymal lesions, only faint, scattered CFP10 signals are detectable in renal tissue, lacking the characteristic glomerular and tubular distribution patterns. Therefore, combining the expression intensity of CFP10 in renal tissue with its characteristic distribution effectively excludes interference from tuberculosis in other organs for the diagnosis of renal tuberculosis, ensuring diagnostic accuracy.

CFP10 is encoded by Rv3874, another gene fragment within the RD-1 region that is deleted in the BCG vaccine, and is present only in pathogenic MTB and a few non-tuberculous mycobacteria (16). Thus, it exhibits high specificity and forms the basis for widely used IGRA tests. Nevertheless, as an immunodiagnostic technique for tuberculosis, IGRA is influenced by the immune status of the patient (17), carrying risks of false positives (e.g., from past infections) and false negatives (e.g., in immunocompromised patients). Furthermore, it cannot distinguish between active tuberculosis and latent infection (18). Direct detection of MTB antigens enables specific diagnosis of active disease independently of host immune responses, with higher accuracy than that of immunological assays. Additionally, CFP10 has been detected in the urine of HIV-infected patients with active tuberculosis, demonstrating its potential as an adjunctive diagnostic tool for tuberculosis (19). Studies addressing the detection of CFP10 in tissues are limited, with only one study reporting its expression in renal tuberculosis tissues, yielding a sensitivity of 63.9% and specificity of 84.60% (20). Our research group previously detected CFP10 in pulmonary and lymph node tissues, achieving sensitivities ranging from 63.16 to 75.00% and specificities from 89.70 to 97.04%. In the current study, the sensitivity of CFP10 detection was 91.84%, and the specificity was 91.89%, demonstrating comparable specificity to previous studies but with a higher sensitivity. A comprehensive analysis of this study and prior studies reveals substantial variability in the sensitivity and specificity of IHC detection of MTB-specific antigens. Potential reasons for this variability include the uneven distribution of MTB and its antigens across different human tissues, which can lead to variations in bacterial and antigen content. Additionally, differences in control groups may impact specificity; for example, certain proteins present in inflammatory or tumor tissues may induce false-positive reactions, thereby reducing specificity. Variations in the antibodies employed across studies may also contribute to inconsistent results. Most critically, the absence of standardized criteria for interpreting IHC staining of tuberculosis-specific antigens significantly affects both sensitivity and specificity. CFP10 is primarily present in *Mycobacterium tuberculosis*, with only a few non-tuberculous mycobacteria capable of producing detectable levels of this antigen. All subjects in this study were excluded from NTM infection via IGRA and *Mycobacterium tuberculosis*-specific gene testing. No significant CFP10 strong positive signals were detected in non-NTM group specimens, suggesting no cross-reactivity with NTM in the CFP10 immunohistochemical detection used here. However, since patients with kidney disease caused by NTM infection were not included in this study, potential cross-reactivity between CFP10 and certain NTM species cannot be entirely ruled out. Future studies should incorporate NTM infection cases to further validate its specificity. CFP10, a secreted antigen encoded by the RD1 region of *Mycobacterium tuberculosis*, circulates via the bloodstream to multiple organs including the lungs, spleen, and lymph nodes. However, this study reveals that its expression in renal tuberculosis tissue exhibits organ-specific distribution characteristics: beyond the multinucleated giant cells surrounding tuberculous granulomas and areas of caseous necrosis, it is also widely present in the glomerular mesangial region, the capillary basement membrane, the cytoplasm of renal tubular epithelial cells, and the renal interstitium. This distribution pattern differs significantly from CFP10 expression in pulmonary tuberculosis and lymph node tuberculosis (where it is primarily localized to alveolar epithelium and pulmonary interstitial granulomas, and to lymphoid follicles and nodular necrosis areas, respectively). Furthermore, in renal tissue from patients with disseminated tuberculosis, CFP10 is detectable but exhibits significantly lower expression intensity compared to primary renal tuberculosis (unpublished data from our research team). This is presumed to correlate with the establishment, proliferation, and sustained CFP10 secretion by *Mycobacterium tuberculosis* within renal tissue during primary renal tuberculosis. In disseminated tuberculosis, renal involvement results solely from hematogenous spread, characterized by low bacterial load and minimal antigen secretion. Thus, the expression site and intensity of CFP10 serve as critical criteria for distinguishing renal tuberculosis from pulmonary tuberculosis and disseminated tuberculosis.

All renal tuberculosis cases included in this study were clinically active renal tuberculosis (confirmed by combined clinical symptoms, imaging, pathogen, and histopathology), and CFP10 immunohistochemical testing detected positive signals in active tuberculosis lesion tissues. In contrast, during latent tuberculosis, *Mycobacterium tuberculosis* remains dormant and scarcely secretes CFP10 antigen, making it difficult to detect CFP10-positive signals in latent renal tuberculosis tissue. This indicates that CFP10 immunohistochemical detection holds specific diagnostic value for active renal tuberculosis but cannot detect latent renal tuberculosis. It may serve as an auxiliary biomarker for clinically distinguishing between active and latent renal tuberculosis.

The comprehensive comparison of the detection efficacy ofIHC, acid-fast staining, and real-time fluorescent PCR revealed that both acid-fast staining and PCR demonstrated 100.00% specificity. The specificity of immunohistochemical detection for CFP-10 was 91.89%, which, although not reaching 100.00%, remains relatively high in clinical practice. The sensitivity of acid-fast staining was extremely low (4.08%), whereas real-time fluorescent PCR demonstrated markedly higher sensitivity at 83.67%. In comparison, the IHC detection sensitivity for CFP-10 was 91.84%, significantly higher than that of acid-fast staining (*p* < 0.001). Although it was also slightly higher than PCR, this difference was not statistically significant (*p* > 0.05). Consistency analysis revealed high concordance between CFP-10 IHC and PCR results (Kappa = 0.653), indicating good agreement between IHC and molecular detection methods. Notably, among the eight qPCR-negative specimens, CFP-10 antigen was still detected in six specimens via IHC. This may be related to MTB DNA degradation, as DNA in tissue specimens, especially paraffin-embedded specimens, is prone to fragmentation and degradation due to formalin fixation and prolonged storage. Additionally, qPCR detection requires a certain bacterial load; additionally, low bacterial counts or residual dead bacteria may result in qPCR-negative outcomes (21). Importantly, MTB antigens, such as CFP-10, are present within MTB cells and widely distributed in the surrounding areas, potentially interfering with macrophage apoptosis and phagosome maturation (22), thereby making them valuable for the supplementary diagnosis of pathogen-negative (qPCR-negative) renal tuberculosis. This study did not directly compare the sensitivity of CFP10 with *Mycobacterium tuberculosis* culture. However, clinical data indicate that the false-negative rate for urine/tissue culture in renal tuberculosis exceeds 70%, while the false-negative rate for acid-fast staining smears reaches 95.92% (only 2 positive cases among 49 specimens in this study). whereas CFP10 immunohistochemistry maintained high detection rates in smear- and PCR-negative renal tuberculosis specimens: 43 of 47 acid-fast smear-negative specimens were CFP10-positive (91.5%), and 6 out of 8 qPCR-negative specimens were CFP10-positive (75%). This indicates that CFP10 immunohistochemistry can effectively compensate for the false-negative limitations of smear, culture, and molecular detection, making it suitable for diagnosing renal tuberculosis in cases with negative pathogen detection results.

This study confirms that CFP10 immunohistochemical detection serves as an effective adjunctive diagnostic tool for renal tuberculosis. This method can be combined with existing clinical diagnostic approaches: Combined with acid-fast staining and real-time fluorescent PCR, it enhances the sensitivity of pathogen diagnosis. When integrated with renal CT/ultrasound and urinary tract imaging, it compensates for the limited specificity of imaging diagnostics. Concurrent use with urine *Mycobacterium tuberculosis* culture shortens the diagnostic cycle, ultimately achieving dual improvements in both sensitivity and specificity for renal tuberculosis diagnosis.

This study still has some limitations. As a retrospective analysis, all specimens used were paraffin-embedded tissues; therefore, bacterial culture could not be performed, resulting in the absence of the gold standard for tuberculosis diagnosis. Future studies could expand the scope to evaluate the diagnostic efficacy of tuberculosis antigens in fresh tissues, particularly in needle biopsy specimens. Second, the control group in this study was relatively homogeneous. Had multiple other disease types been selected as negative controls, the specificity might have differed. Future studies should broaden the types and number of negative controls included to further evaluate diagnostic efficacy. This study included 49 cases of renal tuberculosis. Although the sample size is representative for a single-center retrospective study, it remains relatively small, making it difficult to conduct further subgroup analyses (e.g., differences in CFP10 expression among patients with varying disease duration or treatment statuses). Future prospective, multicenter studies with larger samples are needed to further validate the accuracy and stability of CFP10 immunohistochemical detection in diagnosing renal tuberculosis. The non-TB control group in this study comprised patients with renal clear cell carcinoma. Although all had no history of tuberculosis and negative IGRA results, using kidney tissue from healthy individuals without comorbidities as controls would provide a more intuitive and significant assessment of CFP10 detection specificity for renal tuberculosis. As tumor tissue, renal clear cell carcinoma may exhibit minor non-specific antigen expression induced by inflammatory factors, which partly explains the faint CFP10 positive signals observed in three non-TB specimens in this study. Future studies incorporating healthy kidney tissue controls will further clarify the specificity threshold of CFP10 in diagnosing renal tuberculosis.

## 4 Conclusion

Immunohistochemical detection of CFP10 may serve as a supplementary diagnostic tool for renal tuberculosis, particularly in patients with negative pathogen detection. CFP10 immunohistochemical detection can be used in combination with other diagnostic methods for renal tuberculosis, such as urinary tract imaging, renal imaging, and urinary pathogen detection, to enhance diagnostic sensitivity and specificity. Additionally, this assay provides significant supplementary diagnostic value for active renal tuberculosis cases with negative pathogen detection. However, its clinical application requires validation through large-scale, multicenter studies, incorporating healthy individuals and non-*Mycobacterium tuberculosis* infection cases to further refine specificity assessment.

## Data Availability

The original contributions presented in the study are included in the article/supplementary material, further inquiries can be directed to the corresponding author.
